# Resting-State Time-Varying Analysis Reveals Aberrant Variations of Functional Connectivity in Autism

**DOI:** 10.3389/fnhum.2016.00463

**Published:** 2016-09-16

**Authors:** Zhijun Yao, Bin Hu, Yuanwei Xie, Fang Zheng, Guangyao Liu, Xuejiao Chen, Weihao Zheng

**Affiliations:** ^1^Ubiquitous Awareness and Intelligent Solutions Lab, School of Information Science and Engineering, Lanzhou UniversityLanzhou, China; ^2^Department of Magnetic Resonance, Lanzhou University Second HospitalLanzhou, China

**Keywords:** fMRI, autism, time-varying, functional connectivity, brain state, divergence

## Abstract

Recently, studies based on time-varying functional connectivity have unveiled brain states diversity in some neuropsychiatric disorders, such as schizophrenia and major depressive disorder. However, time-varying functional connectivity analysis of resting-state functional Magnetic Resonance Imaging (fMRI) have been rarely performed on the Autism Spectrum Disorder (ASD). Hence, we performed time-varying connectivity analysis on resting-state fMRI data to investigate brain states mutation in ASD children. ASD showed an imbalance of connectivity state and aberrant ratio of connectivity with different strengths in the whole brain network, and decreased connectivity associated precuneus/posterior cingulate gyrus with medial prefrontal gyrus in default mode network. As compared to typical development children, weak relevance condition (the strength of a large number of connectivities in the state was less than means minus standard deviation of all connection strength) was maintained for a longer time between brain areas of ASD children, and ratios of weak connectivity in brain states varied dramatically in the ASD. In the ASD, the abnormal brain state might be related to repetitive behaviors and stereotypical interests, and macroscopically reflect disruption of gamma-aminobutyric acid at the cellular level. The detection of brain states based on time-varying functional connectivity analysis of resting-state fMRI might be conducive for diagnosis and early intervention of ASD before obvious clinical symptoms.

## 1. Introduction

Autism Spectrum Disorder (ASD) is a lifelong developmental disorder. Children with ASD cannot perform normal social communication, and are characterized by repetitive behaviors and stereotypical interests. Previous studies indicated that impairments in the ASD might reflect deficits in brain network and connectivity (Minshew and Williams, 2007; Vissers et al., 2012; Abbott et al., 2015).

In the neuroimaging analysis, deficits in connectivity have been found to be related to clinical symptoms and behavioral performance in the ASD. Underconnectivity and overconnectivity have been detected in functional connectivity analysis of ASD. In the studies about ASD based on working memory, executive functioning, and response inhibition tasks, the underconnectivity of frontal-posterior connections supported the theory that malfunction of circuitry with underconnectivity could cause deficits in integration of information in the brain at cognitive level (Just et al., 2004; Koshino et al., 2005; Just et al., 2007; Kana et al., 2007). In the resting-state studies, overconnectivity was found to be associated with ASD symptom severity (Keown et al., 2013). In the network analysis of ASD, underconnectivity disturbed the integration of network and overconnectivity damaged the segregation of network (Abbott et al., 2015). Integration within brain networks and segregation between them played an important role in the functional brain maturation (Dosenbach et al., 2010). And in the development (from childhood to early adulthood) of brain, negative function connectivities associated with right superior temporal cortex were increased (Kelly et al., 2009).

Recently, dynamic network analysis was introduced in studies of functional connectivity to identify brain connectivity states. Dynamic network analysis could reveal functional connectivity variability during a scan period, which might be impossible in the traditional network analysis of fMRI data. Dynamic network analysis has found some time-varying characteristics of brain connectivity based on fMRI data during a scan period (Liu and Duyn, 2013; Allen et al., 2014; Monti et al., 2014; Yu et al., 2015). Relevant studies have indicated that metastable states identified by dynamic networks corresponded to stages of consciousness (Calhoun et al., 2014). In addition, dynamic network analysis promoted knowledge of actual sub-network interactions and separation strategies of brain regions (Allen et al., 2014; Yang et al., 2014). In previous studies, dynamic network analysis showed that connectivity state could be shifted in humans with long-term training and experience, such as taxi drivers (Shen et al., 2016). Childhood and adolescence were key stages of brain maturation, and cognitive function networks showed dynamic reorganization in brain maturation (Uddin et al., 2011). And in the development of adolescence, dynamics of brain state was the basis of the development of executive function (Medaglia et al., 2015). ASD might induce deviation of reorganization process from the normal process, and influence connectivity state. In addition, changes of connectivity state in the ASD were less drastic between the resting-state and the tasking-state as compared to typical development (TD) children (Uddin et al., 2015).

Previous functional connectivity fMRI studies showed that overconnectivity and underconnectivity were the major forms of abnormal connectivity in the ASD. To the best of our knowledge, the relationship between connectivity strength and time-varying functional connectivity states in the ASD based on fMRI has not yet been reported. We hypothesized that ASD could influence time-varying functional connectivity states through affecting distribution of connectivity strength and influence the connectivities related to social function. To investigate the influence of ASD on brain connectivity states, we performed group independent component analysis (GICA) and dynamic network analysis on fMRI data of ASD and TD children. GICA can extract spatial distribution of functional regions in the brain.

## 2. Materials and methods

### 2.1. Participants and functional MRI data acquisition

Data of participants were obtained from open accessed dataset collected by NYU Langone Medical Center, a collection site of Autism Brain Image Data Exchange I(ABIDE I) (Di Martino et al., 2014). The site includes 79 (7.1–39.1 years) ASD and 105 TD (6.5–31.8 years) children. The criteria of included subjects are:

1. male
2. scores of full intelligence quotient (FIQ, estimated by the fourth subtests of the Wechsler Abbreviated Scale of Intelligence, WASI-IV) above 85
3. right-handedness
4. aged 7–18 (not including 18 years old)

TD children were matched with ASD children for age, gender, handedness, FIQ score, and head motion (*P*-values of the rigid 6 using two-sample *t*-test were 0.7654, 0.8762, 0.2053, 0.6026, 0.5831, and 0.6601, respectively). The detailed demographic information of participants is presented in Table 1. BOLD fMRI data of each participant were acquired with a whole-brain echo planar imaging (EPI) sequence and interleaved slice acquisition (TR = 2 s, TE = 15 ms, flip angle = 90°, slice thickness = 4 mm, FoV = 240 mm, 180 volumes) on a 3T Allegra scanner. Data collections were approved by local IRB of the site, and all data were anonymized. More detailed information is available at http://fcon_1000.projects.nitrc.org/indi/abide/.

**Table 1:** **Demographic information of the participants**.

	**TD**	**ASD**	***P*-value**
*N*	44	31	-
Age (*Mean* ± *SD*)	12.46 ± 3.1	11.51 ± 2.64	0.1693
Gender	Male	Male	–
Handedness	Right	Right	–
Handedness Score (*Mean* ± *SD*)	62.07 ± 22.82	63.52 ± 23.88	0.7914
FIQ Score (*Mean* ± *SD*)	113.14 ± 12.32	112.52 ± 15.87	0.8495
ADI-R Social Total A (*Mean* ± *SD*)	–	18.77 ± 4.66	–
ADI-R Verbal Total BV (*Mean* ± *SD*)	–	15.26 ± 3.84	–
ADI RRB Total C (*Mean* ± *SD*)	–	5.74 ± 2.61	–
ADI R Onset Total D (*Mean* ± *SD*)	–	2.94 ± 1.34	–
ADOS Module	–	3	–
ADOS Total (*Mean* ± *SD*)	–	11.52 ± 4.41	–
ADOS Communication (*Mean* ± *SD*)	–	3.41 ± 1.74	–
ADOS Social (*Mean* ± *SD*)	–	8.11 ± 3.04	–
ADOS Stereo Behavior (*Mean* ± *SD*)	–	2.67 ± 1.95	–

*ADI-R, Autism Diagnostic Interview-Revised; ADOS, Autism Diagnostic Observation Schedule; Subjects evaluated by ADOS module 4 were excluded when we calculated mean and SD of ADOS scores*.

### 2.2. Data preprocessing

Resting-state fMRI raw data were preprocessed by Data Processing Assistant for Resting-State fMRI (DPARSF) (Chao-Gan and Yu-Feng, 2010) based on Statistical Parametric Mapping (SPM8). The procedure of preprocessing included removal of first 10 image volumes, realignment, time-slicing and head motion correction, normalization into Montreal Neurological Institute (MNI) standard space, and spatially smoothened by a full-width at half-maximum of 6 mm. All image volumes were aligned to the first volume for each participant in the realignment. In the head motion correction, head motion parameters were estimated according to Friston 24-Parameter Model (Friston et al., 1996). In the normalization, fMRI data were spatially normalized to the MNI EPI template.

### 2.3. Independent component analysis

**Figure 1:**
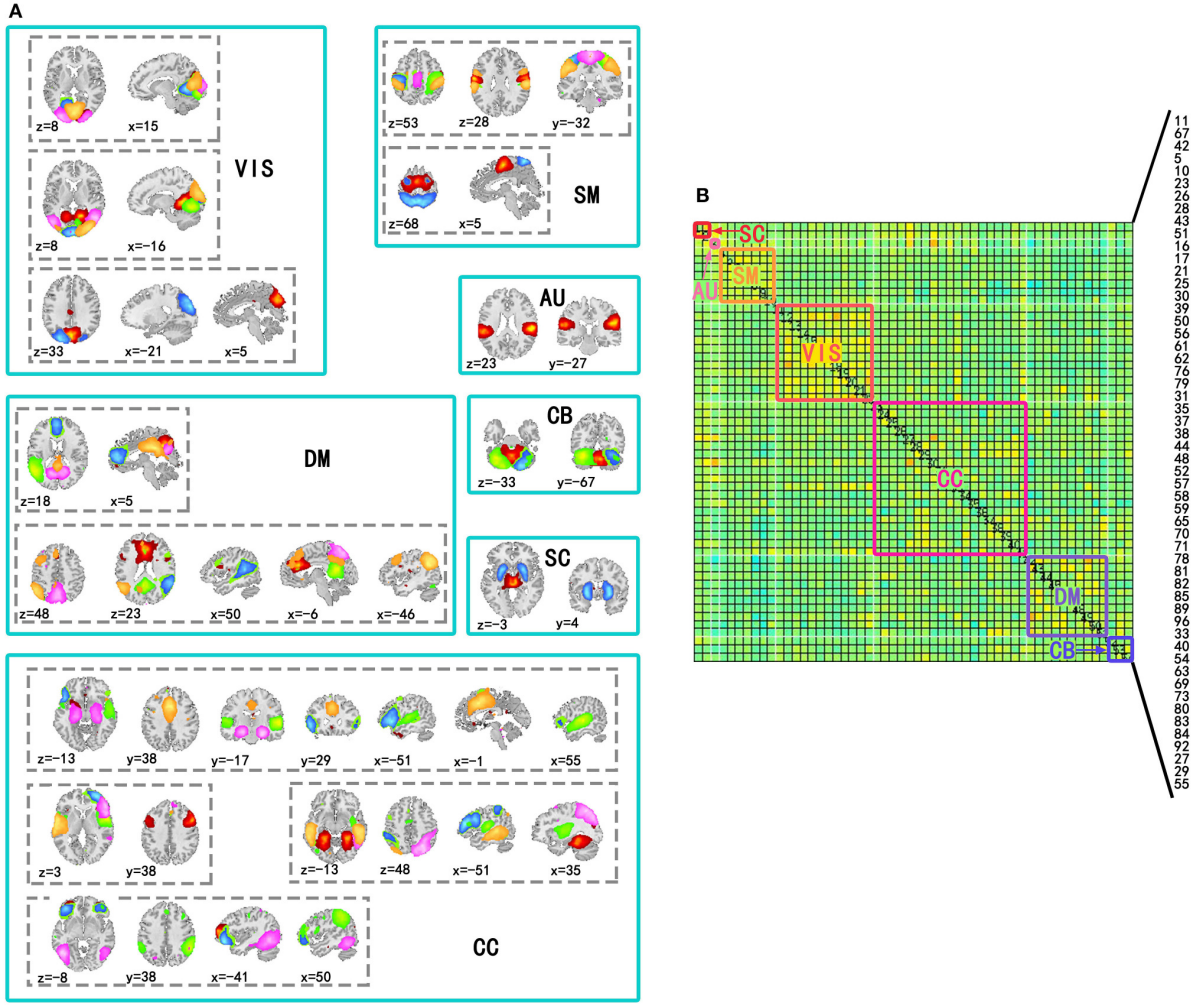
**Spatial distribution of components used to construct time-varying networks and seven subnetworks in the covariance matrix**. **(A)** Spatial distributions of components involved in the same subnetwork were in a solid line box and spatial distributions of the same components were in a dotted line box. In a dotted line box, colors of regions corresponded to ICs. **(B)** Subnetworks were labeled by solid line boxes and corresponding texts in a covariance matrix of a state. The number of the components in the matrix has been labeled in the figure (11, 67, 42, 5, 10, 23, 26, 28, 43, 51, 16, 17, 21, 25, 30, 39, 50, 56, 61, 62, 76, 79, 31, 35, 37, 38, 44, 48, 52, 57, 58, 59, 65, 70, 71, 78, 81, 82, 85, 89, 96, 33, 40, 54, 63, 69, 73, 80, 83, 84, 92, 27, 29, and 55).

Independent component analysis was performed on the preprocessed fMRI data by GIFT v3.0a using Infomax algorithm (Bell and Sejnowski, 1995), and the order of ICA model was 100. Before performing ICA algorithm, fMRI data dimension reduction was performed by Principal Component Analysis (PCA). The reliability of independent components (ICs) was evaluated by repeating the algorithm 25 times in ICASSO (Himberg and Hyvärinen, 2003). According to spatial distribution of ICs in previous studies (Allen et al., 2014), 54 ICs in seven sub-networks were kept for the following analysis. The seven sub-networks were subcortical (SC), auditory (AU), visual (VIS), somatomotor (SM), cognitive control (CC), default mode (DM), and cerebellar (CB) networks (Figure 1). The other components were related to movement or physical according to their spatial distributions, so they were not included in this study.

### 2.4. Calculation of time-varying connectivity and k-means clustering

The selected ICs were defined as regions of interest (ROIs) to construct the networks. Linear, quadratic, and cubic trends of the time courses extracted according to the ROIs were removed, and six realignment parameters were regressed out. Then, the regressed time courses were depicted by 3DDESPIKE to remove the outliers, and filtered with a high cutoff frequency of 0.15 Hz according to the previous study (Allen et al., 2014).

Time-varying functional connectivity was calculated based on segmented time courses in 148 windows created by a tapered window [a rectangle (width = 22 TRs) with a Gaussian (=3 TRs)] sliding in steps of 1 TR. By calculating Pearson's correlation coefficient (functional connectivity) of all possible ROI pairs in the same sliding window, we constructed covariance matrices (54 × 54) of each subject. In addition, we used the graphical LASSO (a shrinkage and selection method for linear regression) to evaluate the log-likelihood of covariance matrices, and regularized matrices after evaluating with L1-norm penalty to control sparsity (Friedman et al., 2008).

To determine the connectivity states, covariance matrices of ASD and TD were clustered by k-means clustering algorithm based on Manhattan distance. Clustered centroid matrices were covariance matrices of connectivity states. We used gap, elbow and Calinski Harabasz to estimate the optimal cluster number. However, optimal cluster number of these methods was two and this was improper. So clustering was performed at *k* = 2 to 20, and repeated 150 times per *k*-value. The effectiveness of the states in the ASD and NC was determined upon the span of states in windows number. In this study, reliable state in the ASD and NC covered at least 10 windows; otherwise, state (covered < 10 windows) was unreliable. Mean dwell time (MDT) was calculated at each *k*-value. MDT was the average number of windows that were continuous on the time distribution and classified as the same state, representing the duration of each state.

### 2.5. Statistical analysis

Two-sample *t*-test was used to compare the MDTs of ASD and TD children. To detect the differences of connectivity in each connectivity state, subjects with effective state were included in the two-sample *t*-test for the median covariance matrices of each state. In each *k*-value, median covariance matrices were identified by Manhattan distance priority and tested with the two-sample *t*-test, with a threshold (*p* < 0.001) to identify connectivities with differences. Times of different connectivity at each state for all *k*-values were aggregated. Connectivity with frequency of occurrence ≥5 times was included in the results.

### 2.6. Connectivity strength discretization

The absolute values of connectivity strength in all covariance matrices for all *k*-values were divided into three levels (1, 2, and 3) by discretization method based on average and standard deviation of TD (0.1828 ± 0.1363) and ASD (0.1698 ± 0.1322) separately. In this study, the connectivity of first level was defined as weak connectivity and last level as strong connectivity. We calculated the percentages for three types of connectivity in all clusters to determine changes in the number of connectivity with different strength in the ASD.

## 3. Results

**Figure 2:**
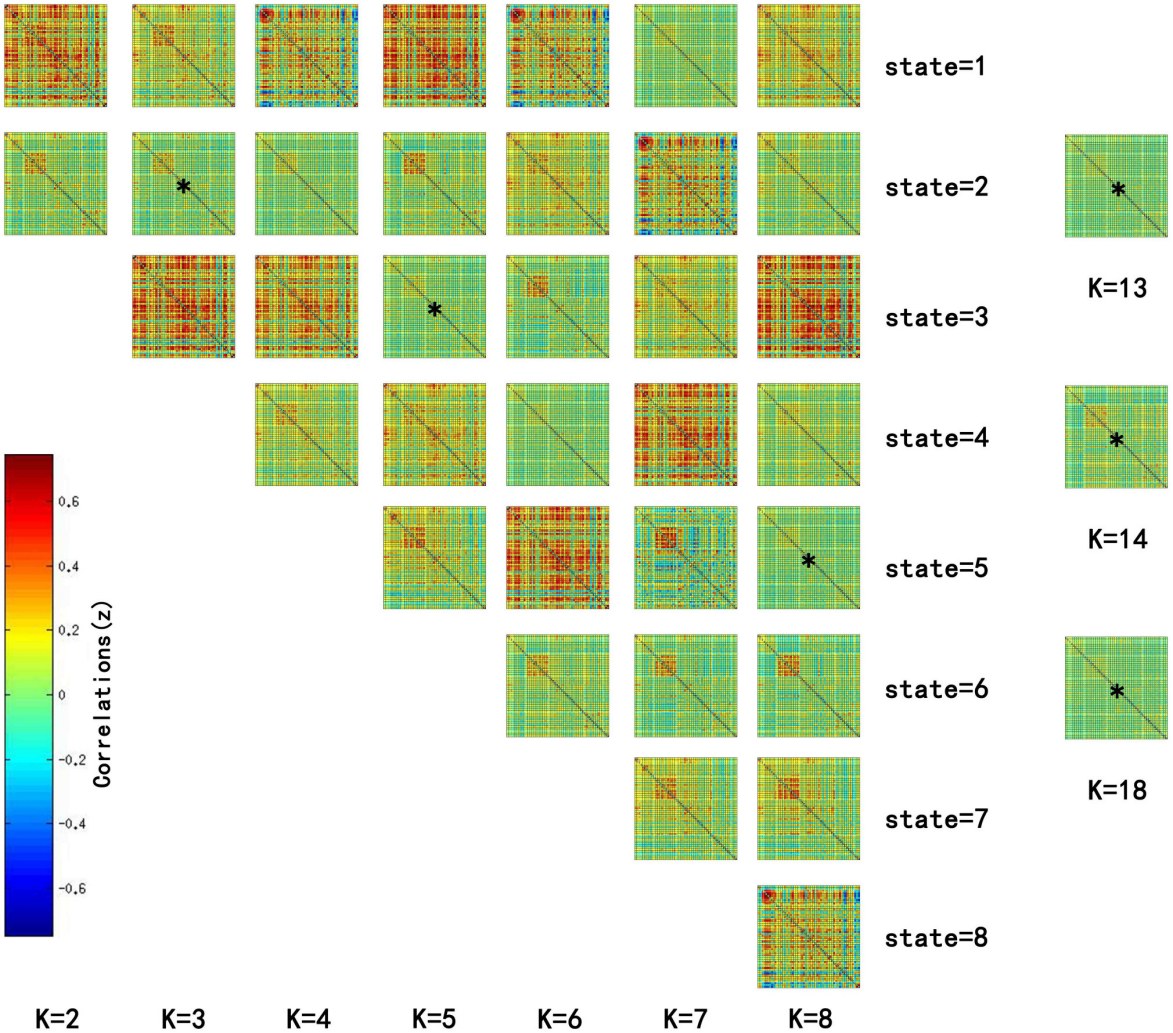
**Matrices of all states at ***k*** = 2 to 8 and clustered centroid matrices with significantly different MDTs (***p*** < 0.05)**. *K* was the number of clusters in k-means clustering. This figure showed all centroids of states at *k* = 2 to 8 and centroid matrices with significantly different MDTs at *k* = 13, 14, and 18. MDTs of some states (marked with stars) in the figure were significantly different when values of k were 3 (*T* = 2.1733, *p* = 0.0330 < 0.05), 5 (*T* = 2.1247, *p* = 0.0370 < 0.05), and 8 (*T* = 2.2591, *p* = 0.0269 < 0.05). *T*- and *p*-values were calculated by two-sample *t*-test. In addition, state matrices with significantly different MDTs were marked with star when k were 13 (*T* = 2.6400, *p* = 0.0101 < 0.05), 14 (*T* = 2.3359, *p* = 0.0222 < 0.05), and 18 (*T* = 2.0610, *p* = 0.0429 < 0.05).

**Figure 3:**
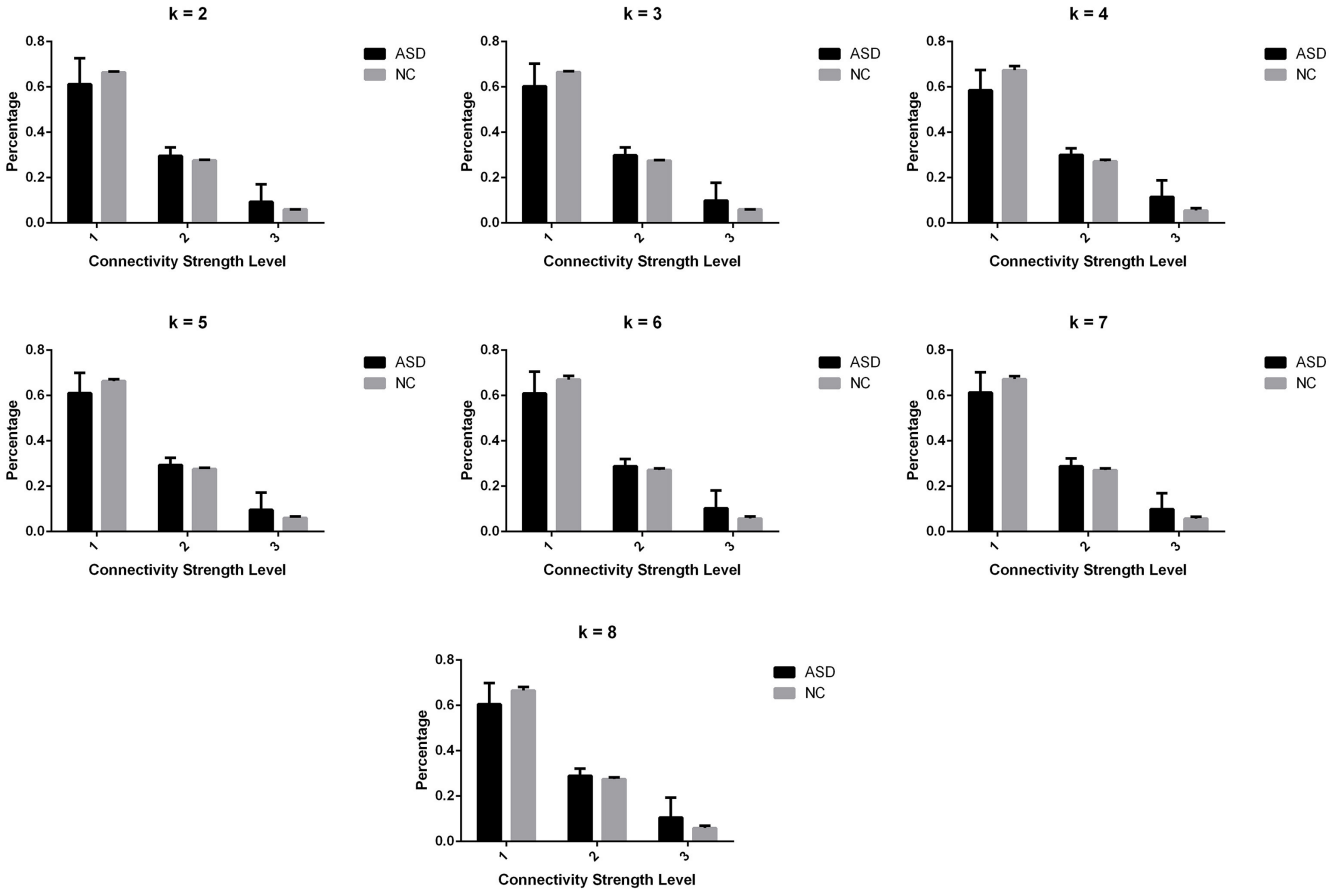
**Means and standard deviations of percentages for three types of connectivity at ***k***-values from 2 to 8**.

**Figure 4:**
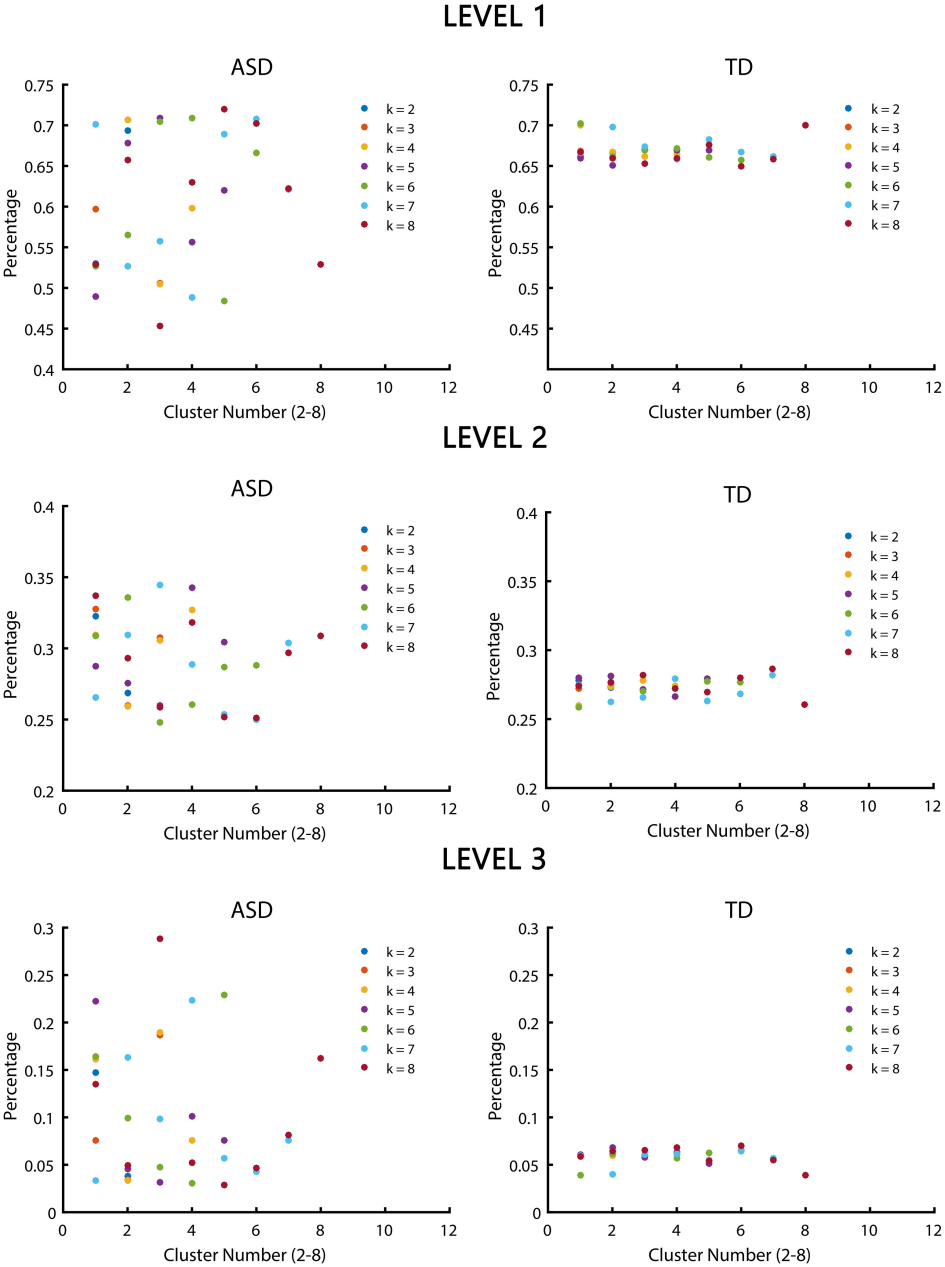
**Scatter diagrams of percentages for three types of connectivity at ***k***-values from 2 to 8**. *K* was the number of clusters in the clustering process. Level 1, level 2, and level 3 represented three types of connectivity.

Significant differences in the MDTs were found when the *k*-values of k-means clustering were 3, 5, 8, 13, 14, and 18. Functional connectivity of ASD children showed weak connectivity for a longer time as compared to TD children according to the *T*-values. Figure 2 shows detailed information of the clustered centroid matrices with significantly different MDTs. Table 2 shows the percentage of three connectivity strength levels at *k*-values with significantly different MDTs. The clusters with maximum ratio of weak connectivity were with significantly different MDTs when *k* was 3, 5, and 8. However, unreliable clusters existed when *k*-values were >8. Figure 3 shows means and standard deviations of percentages for three types of connectivity when *k*-values were from 2 to 8. Figures 3, 4 indicate less means of weak connectivity in the ASD as compared to TD. Figure 4 also shows that the fluctuation range of percentages for three types of connectivity in the ASD was greater as compared to TD.

**Table 2:** **Percentages for three types of connectivity in all clusters of different ***k***-values with significantly different MDTs**.

**Cluster number (k)**	**Percentage of 1 (weak connectivity)**		**Percentage of 2**		**Percentage of 3 (strong connectivity)**	
	**ASD**	**NC**	**ASD**	**NC**	**ASD**	**NC**
3	0.5967	0.6687	0.3275	0.2720	0.0757	0.0591
	**0.7063**	0.6661	0.2598	0.2738	0.0338	0.0600
	0.5059	0.6618	0.3073	0.2782	0.1866	0.0599
5	0.4896	0.6593	0.2876	0.2796	0.2226	0.0610
	0.6784	0.6512	0.2758	0.2809	0.0457	0.0678
	**0.7085**	0.6704	0.2596	0.2716	0.0318	0.0579
	0.5566	0.6695	0.3424	0.2666	0.1008	0.0638
	0.6196	0.6691	0.3041	0.2795	0.0761	0.0512
8	0.5285	0.6675	0.3368	0.2740	0.1345	0.0584
	0.6573	0.6590	0.2933	0.2764	0.0493	0.0645
	0.4531	0.6531	0.2585	0.2817	0.2883	0.0651
	0.6296	0.6597	0.3180	0.2722	0.0522	0.0680
	**0.7198**	0.6758	0.2518	0.2698	0.0282	0.0542
	0.7018	0.6500	0.2513	0.2801	0.0468	0.0697
	0.6216	0.6587	0.2970	0.2864	0.0813	0.0547
	0.5290	0.7002	0.3089	0.2605	0.1620	0.0391

*Bold percentages were of weak connectivity in clusters with significantly different MDTs at different k-values*.

**Figure 5:**
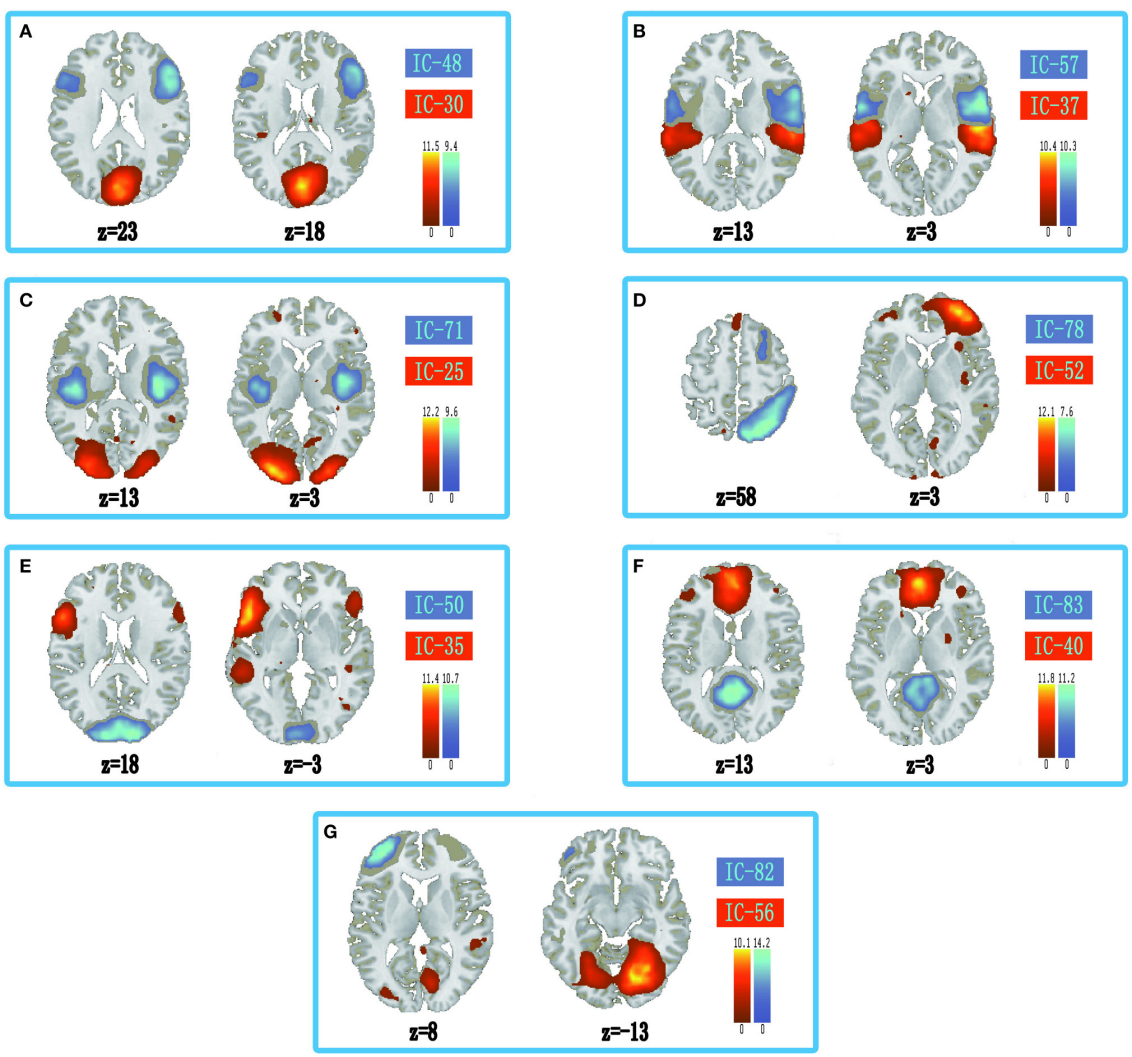
**Spatial distribution of independent components linked by abnormal connectivities**. The connectivity between independent components in each panel occurred more than five times. **(A)** Red-left cuneus (IC 30), blue-right inferior frontal gyrus: orbital part (IC 48); **(B)** Red-right superior temporal gyrus (IC 37), blue-right rolandic operculum (IC 57); **(C)** Red-left middle occipital gyrus (IC 25), blue-right insula (IC 71); **(D)** Red-right middle frontal gyrus (IC-52), blue-right inferior temporal gyrus (IC 78); **(E)** Red-inferior frontal gyrus: orbital part (IC 35), blue-left calcarine sulcus (IC 50); **(F)** Red-left superior middle frontal gyrus (IC 40), blue-precuneus/posterior cingulate gyrus (IC 83); **(G)** Red-right fusiform gyrus (IC 56), blue-left middle frontal gyrus (IC 82).

The information of abnormal connectivities is presented in Table 3, and the ICs connected by these connectivities are shown in Figure 5, Table 4. The ICs were distributed in cognitive control (ICs: 35, 37, 48, 52, 57, 71, 78, 82), visual (ICs: 25, 30, 50, 56), and default mode (ICs: 40, 83) networks. These ICs mainly involved MOG.L, CUN.L, frontal lobe (ORBinf.L, IFGoperc.R, MFG.R, and SFGmed.L), right temporal lobe (STG.R and ITG.R), ROL.R, FFG.R, CAL.L, INS.R, and PCUN/PCG in spatial distribution.

**Table 3:** **The abnormal connectivities with ≥ 5 times recurrence in the ASD**.

**No**.	**Related components**	**Frequency of of occurrence**	**Increase or decrease**
1	30, 48	10	Increase
2	37, 57	10	Increase
3	25, 71	6	Increase
4	52, 78	6	Decrease
5	35, 50	5	Decrease
6	40, 83	5	Decrease
7	56, 82	5	Decrease

**Table 4:** **Peak values distribution of the independent component spatial maps**.

**Independent Component**	**Peak MNI coordinate**			**Peak intensity (*z*-score)**	**Brain regions**
	***X***	***Y***	***Z***		
25	−27	−96	3	11.3693	Left middle occipital gyrus (MOG.L)
30	0	−81	15	10.9039	Left cuneus (CUN.L)
35	−48	21	−6	11.0173	Left inferior frontal gyrus, orbital part (ORBinf.L)
37	54	−21	3	9.7456	Right superior temporal gyrus (STG.R)
40	−3	54	3	11.3276	Left superior frontal gyrus, medial (SFGmed.L)
48	54	21	33	8.7204	Right inferior frontal gyrus, opercular part (IFGoperc.R)
50	0	−99	6	10.4117	Left calcarine fissure and surrounding cortex (CAL.L)
52	33	57	3	11.2562	Right middle frontal gyrus (MFG.R)
56	18	−78	−9	9.7335	Right fusiform gyrus (FFG.R)
57	57	3	3	9.5117	Right rolandic operculum (ROL.R)
71	39	−6	6	9.1991	Right insula (INS.R)
78	21	−72	54	7.2923	Right inferior temporal gyrus (ITG.R)
82	−36	51	9	13.4001	Left middle frontal gyrus (MFG.L)
83	−3	−63	15	10.8304	Precuneus / Posterior cingulate gyrus (PCUN/PCG)

## 4. Discussion

The connectivity state in the brain is flexible, which might correspond to diversity of human cognitive functions. Brain connectivity state could change with maturity of the brain, environmental stimulus, and some developmental disorders. And cognitive and behavioral flexibility have been found decreased in the ASD. Also, damages in the connectivities within and between sub-networks (such as default mode, salience, and executive control networks) have been detected in the studies of atypical connectivity patterns and maturation of the ASD (Washington et al., 2014; Abbott et al., 2015).

In the current study, we used time-varying connectivity analysis to detect the impairments of connectivity states in the ASD at resting state. MDTs of brain states with weaker connectivity were abnormal in the ASD (Figure 2). Also, ratios of connectivity with different strengths changed more drastically in the ASD (Figures 3, 4). Drastic changes of connectivity strength might mask task-evoked connectivity changes, and make the brain states undifferentiated (Rubenstein and Merzenich, 2003; Uddin et al., 2015).

In our study, ASD showed more divergent connectivity strength of brain state than TD (Table 2, Figure 2). In the brain, cognitive function depended on connections of specific brain areas. For example, social cognition was related to the prefrontal cortex, the precuneus/posterior cingulate, the hippocampus, the anterior temporal lobes, the posterior superior temporal sulcus and temporo-parietal junction, the fusiform gyrus, the left inferior frontal gyrus, and the anterior insula (Gotts et al., 2012). Hence, some task-evoked functional connectivities were consistent. Based on the consistency, cognitive function state of the brain could be distinguished by whole-brain connectivity patterns (Shirer et al., 2012). Under the same cognitive function, brain states of ASD might diverge from TD due to the disturbed convergence of functional connectivity in the ASD. Also, atypical connectivity patterns of response inhibition were found in previous studies (Kana et al., 2007; Daly et al., 2014).

The abnormal connectivity states may be macroscopical reflection of the excitatory/inhibitory imbalance at the cellular level (Thatcher et al., 2009; Coghlan et al., 2012). In the ASD, stereotypical behavior was found to be related to abnormal gamma-aminobutyric acid (GABA) signaling (Chao et al., 2010). A previous study reported that increased inhibition or decreased excitation at the cellular level might be noise for brain spontaneous activity measured by fMRI, and interfere with neural synchronization of brain in the ASD (Dinstein et al., 2011). The disrupted excitatory/inhibitory balance in the nerve cells might result in disruption of the connectivity on the macro scale, because functional connectivity is a measure of the synchronization between discrete brain regions (Dinstein et al., 2011). In addition, increased connectivity between subcortical and cortical cortices from fMRI studies have been observed as well as decreased ratio of GABA to creatinine in the cerebellum and the primary sensory and motor cortices in the ASD (Gaetz et al., 2014; Rojas et al., 2014; Cerliani et al., 2015). The locations of abnormal connectivity and disrupted GABA signaling were consistent, which might indicate that the abnormal brain activities resulted from aberrant GABA signaling in the ASD. Aberrant connectivity status in fMRI might play an important role in the diagnosis of ASD without obvious clinical symptoms.

In the ASD, several cognitive circuits in the brain were aberrant, such as circuits related to visual control, working memory, inhibitory control, emotion processing, face recognition, etc. Some aberrant connectivities in the ASD were connected to social brain, which were the discrete brain regions dominating social cognition (Frith and Frith, 2007; Gotts et al., 2012). In our results, ASD showed decreased connectivity between posterior and frontal regions in DMN (PCUN/PCG and SFGmed.L, Figure 5F). The decreased connectivity was also associated with social deficits, and hampered the ability to maintain a conversation, make eye contact, and perform the pragmatics of language (Assaf et al., 2010; von dem Hagen et al., 2013). In addition, abnormal connectivities in the ASD were also found in and between cognitive control (ORBinf.L, IFGoperc.R, INS.R, MFG, STG.R, ITG.R, and ROL.R) and visual networks (left middle occipital gyrus, left cuneus, left calcarine sulcus, and right fusiform gyrus) in our results. The anterior cingulate cortex, ventrolateral prefrontal cortex, dorsolateral prefrontal cortex, and parietal cortex were associated with cognitive control (Solomon et al., 2014). In the human brain, V1 of visual cortex lies in calcarine sulcus, and motion area of visual cortex is located in the inferior temporal sulcus (Orban et al., 2004). Fusiform gyrus was a key region in face recognition and other social functions (Haxby et al., 2000; Liu et al., 2015). Moreover, CUN was related to control of visual attention and refreshing information in working memory (Makino et al., 2004; Roth and Courtney, 2007; Souliéres et al., 2009). Our results showed decreased connectivity related to visual network in the ASD (Figures 5A,C,E,G). In addition, several studies indicated that frontal lobe and right anterior insula played an important role in inhibitory control (Cai et al., 2014; Daly et al., 2014; Shafritz et al., 2015). Our results showed significantly abnormal connectivities linked to medial prefrontal cortex (MPFC) and superior temporal gyrus in the ASD (Figures 5B,D), which might be related to aberrant activation levels in these brain regions and abnormally implicit emotion processing in the ASD (Kana et al., 2016). These results showed that abnormally activated brain regions induced by tasks might be aberrant at resting-state, which might display targeted behavior modification in the ASD before clinical symptoms.

The disruption of excitation and inhibition balance at connectivity or circuit level might contribute to clinical symptoms in the ASD. Time-varying connectivity analysis in resting-state fMRI can identify the influence of excitation and inhibition balance on whole brain connectivity state, and abnormal connectivity at resting-state in the ASD. However, underlying pathological mechanisms of ASD relied on the study of neurotransmitters in neurons, and relationship between abnormal connectivity and cognitive function might hinder tasking-state neuroimaging and electrophysiological study. In addition, the volume or scan time of samples was relatively small in this study. Prolonging the scan time could capture more accurate metastable states and data dependence of the method affected the universal application of the conclusion. Our study might reflect some characteristics of time-varying functional state in the ASD and the differences of connectivity states in dynamic network analysis between ASD and TD groups might suggest the imbalance between excitation and inhibition.

## Author contributions

ZY, BH, and YX conceived and designed the experiments. ZY, YX, and XC organized and analyzed the raw data. ZY, YX, and GL participated in the statistical analysis and interpretation of data. ZY and YX wrote the article, and BH, FZ, and WZ revised the manuscript.

## Funding

This work was supported by the National Basic Research Program of China (973 Program) (No. 2014CB744600, to BH); the National Natural Science Foundation of China (No. 60973138 and 61003240, to BH); and the International Cooperation Project of Ministry of Science and Technology (No. 2013DFA11140, to BH).

### Conflict of interest statement

The authors declare that the research was conducted in the absence of any commercial or financial relationships that could be construed as a potential conflict of interest.
